# Supplementary material for: Experimental phage evolution results in expanded host ranges against antibiotic resistant Klebsiella pneumoniae isolates
Source: Nat Commun. 2025 Nov 19;16:9903. doi: 10.1038/s41467-025-66062-7 (PMC12630596; doi:10.1038/s41467-025-66062-7)
Supplement: Supplementary file 1 — Supplementary Information [file 41467_2025_66062_MOESM1_ESM.pdf]

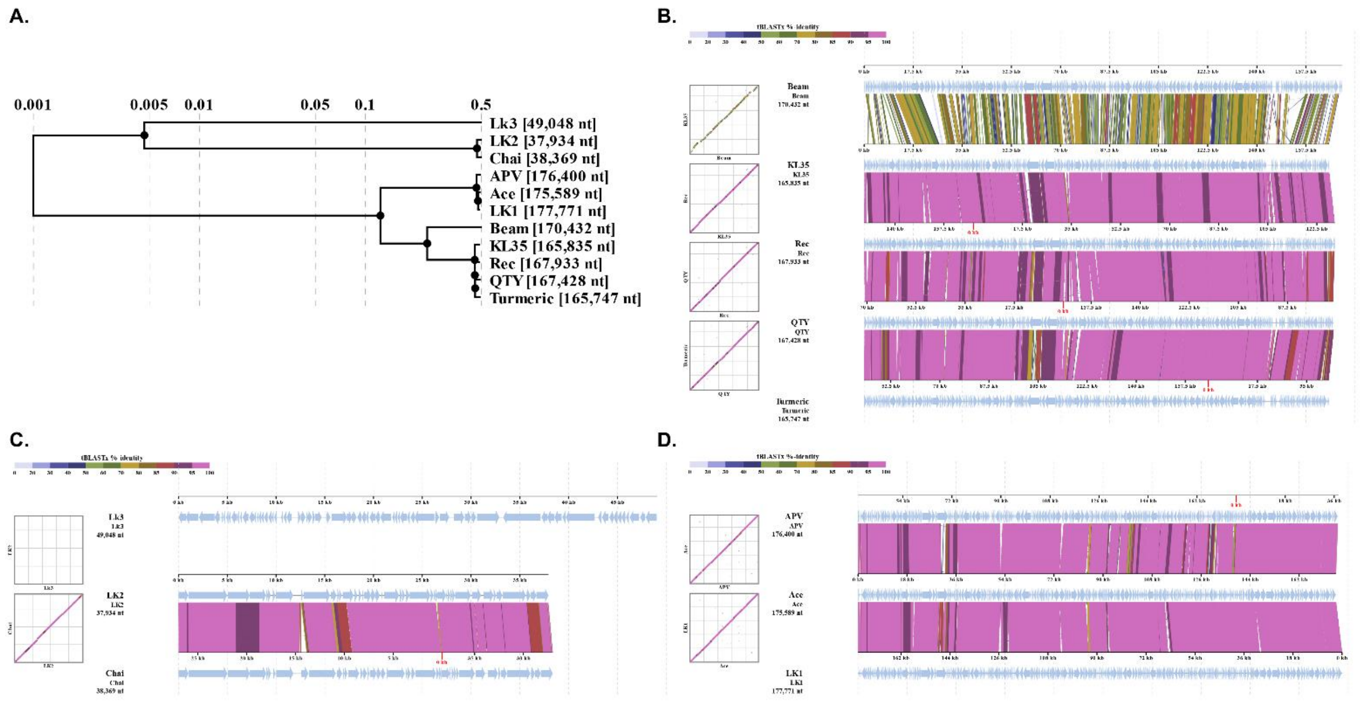

Figure S1. Comparative genomics of phages used in this study. Proteomic tree of all phages used in the study was generated using ViPTree (A). Amino acid-resolved genome alignments for the four phylogenetic clades were generated using DiGAlign (B-D).

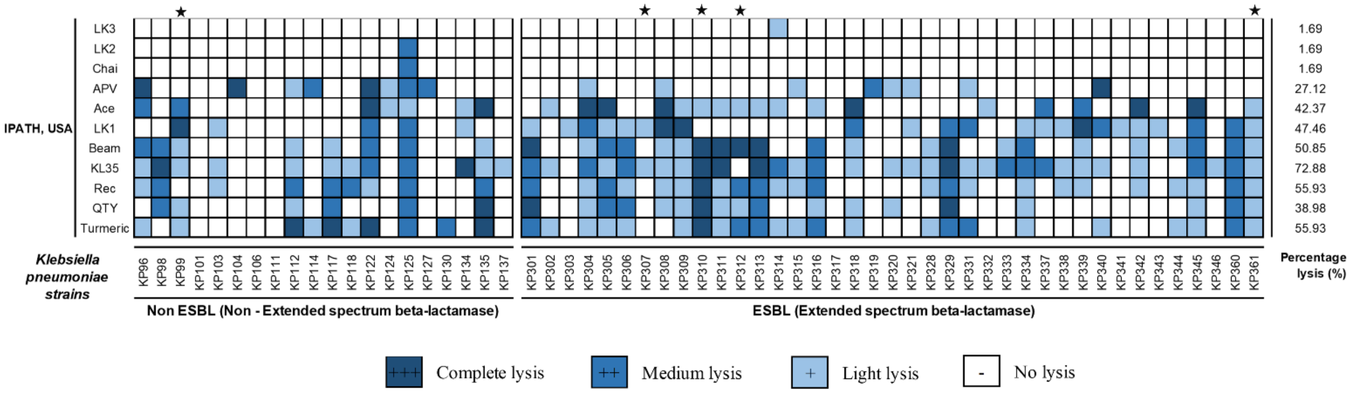

Figure S2. Host range and total percentage killing of the phages against the collection of 59 clinical strains of *Klebsiella pneumoniae*. Phage lysate (5 µl) with 10^7 titer were spotted on a bacterial lawn. After overnight incubation, the plates were examined for lysis & no lysis. The dark blue boxes indicate complete lysis, medium to light blue boxes indicate medium to light lysis and boxes with no color represent no lysis. The isolates were grouped based on bacteria that expressed Extended spectrum beta-lactamases (ESBLs). CRE (Carbapenem Resistant Enterobacteriaceae) isolates are indicated with a star.

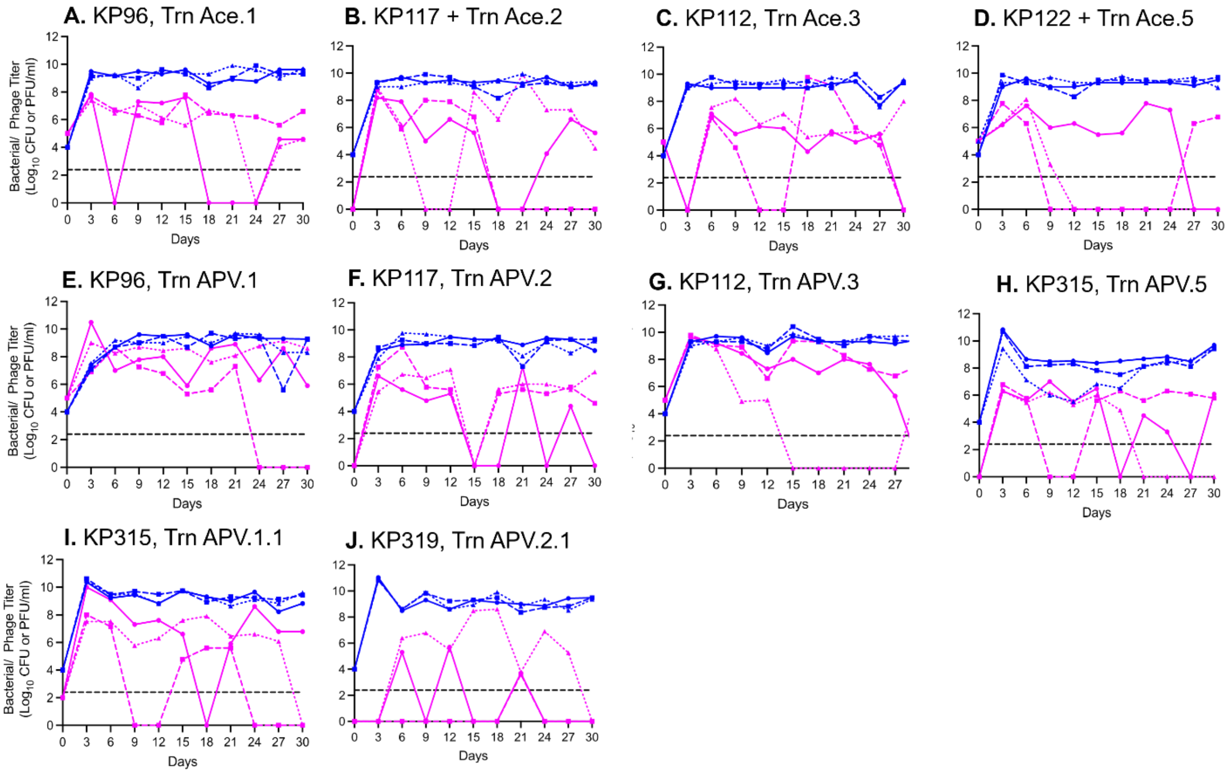

Figure S3. Phage and bacteria abundances during coevolution. The data here represent the population dynamics of phage and bacteria during 30 consecutive days of coculturing different phage and bacterial genotypes (Panels A-J). This was determined based on estimated plaque forming units (PFU) and colony forming units (CFU) at every day 3 of coculturing. The data shown in blue colored lines correspond to bacterial density while the data shown in magenta-colored lines correspond to phage density (Panels A-J). The different line types (solid - R1, dotted - R2, dashed - R3) correspond to different biological replicates.

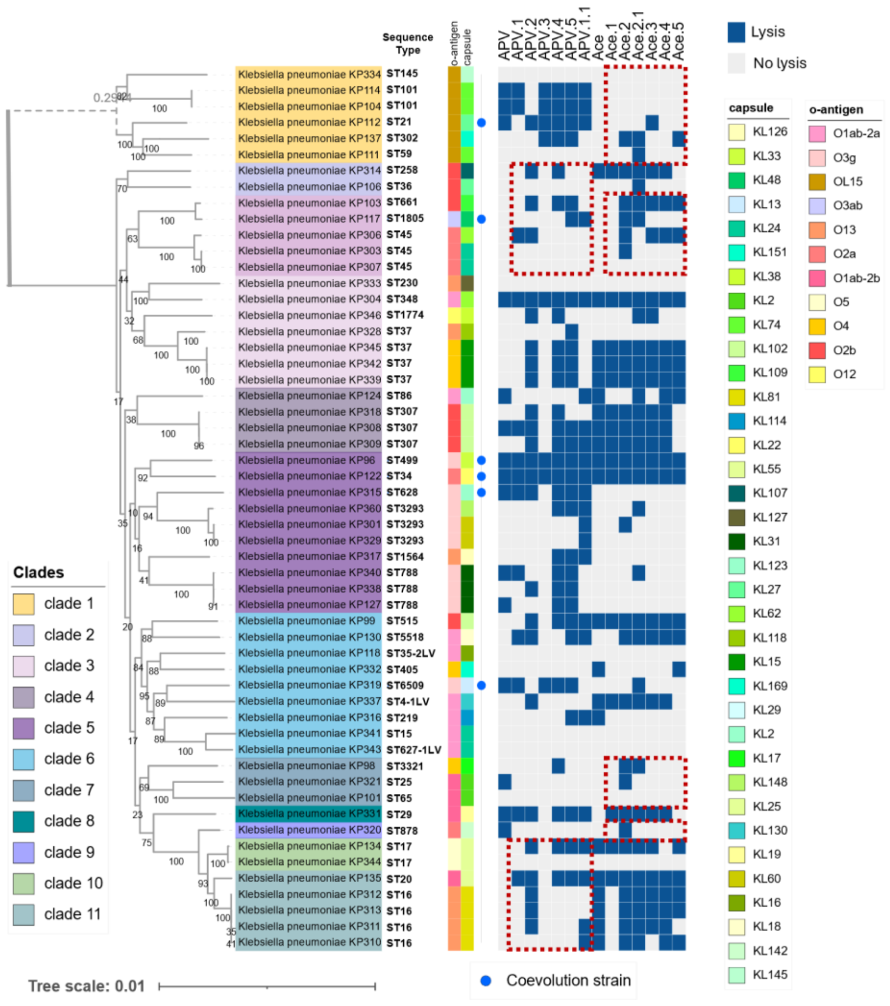

Figure S4. Genomic characterization of clinical strains of *Klebsiella pneumoniae.* Phylogenetic tree of the whole genomes using a codon tree method. Bootstrap values are shown in black. Branch distances are shown in grey. Strains used in the coevolution experiments are shown with a blue circle. Eleven distinctive clades were determined. Capsule and o-antigen groups were determined using Kaptive. An infectivity matrix of ancestral and coevolved phages is shown. Regions where host-range expansion to clades not infected by the ancestral phage are shown in red dotted regions.

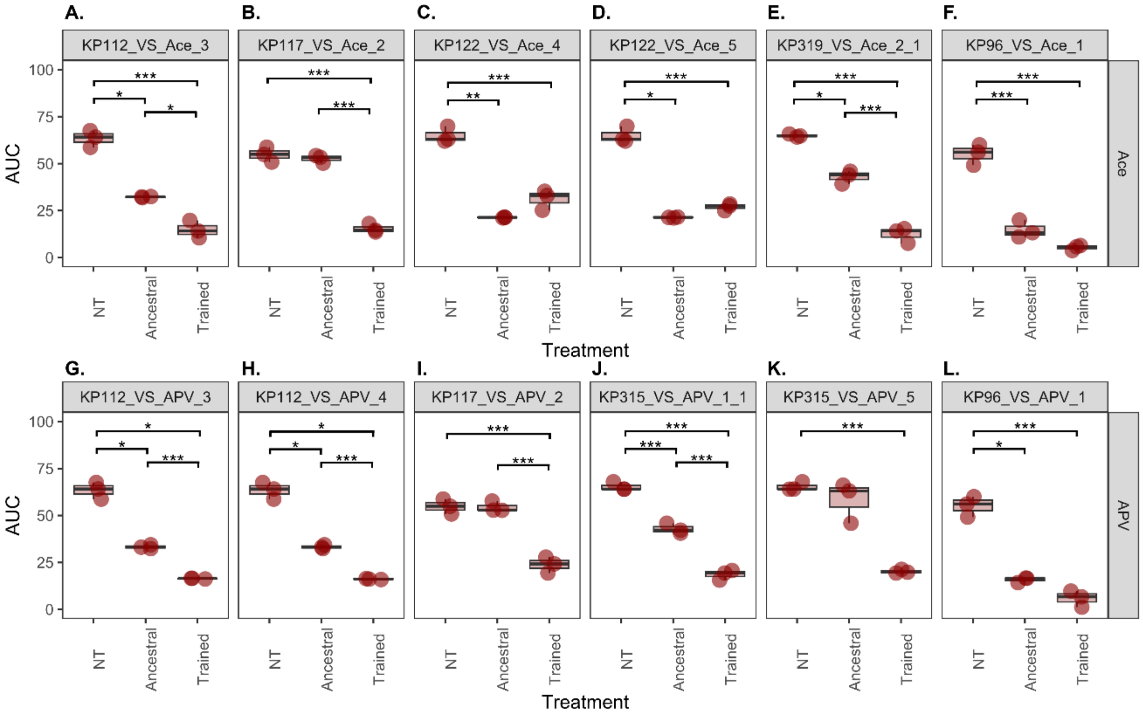

Figure S5. Area Under the Curve (AUC) of growth dynamics of coevolved hosts in the presence of trained vs untrained phages at an MOI of 1 (Panels A-L). NT represents no treatment, Ancestral represents isolates grown in the presence of the ancestral versions of the phages and Trained represents isolates grown in the presence of the trained phages. Three biological replicates were performed for each experiment. Statistical significance based on AUC analysis is represented by the '*' above in each panel, where the brackets indicate which biological replicates from which experiments are being compared. A single * represents p<0.05, ** represents p<0.01, and *** represents p<0.001.

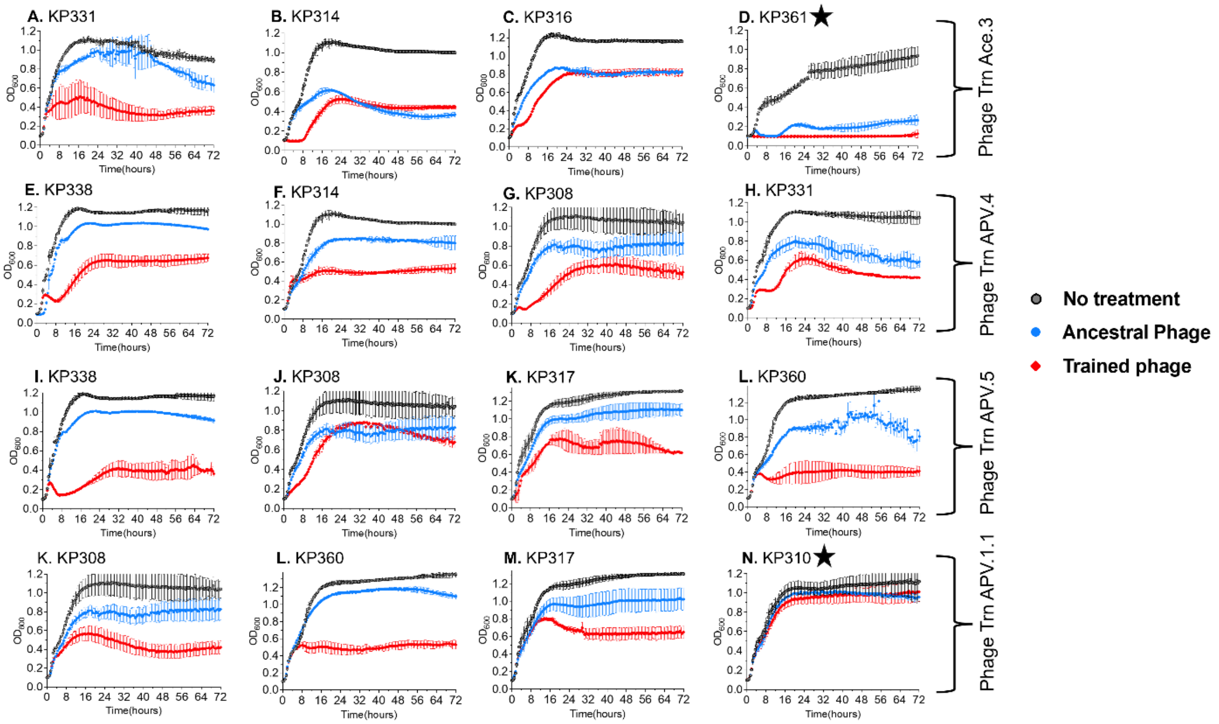

Figure S6. Growth dynamics of ESBL Klebsiella isolates in presence of trained vs untrained phages at MOI of 1 (Panels A-N). Bacterial growth was monitored for 72 hours by measuring the OD600 every 15 minutes in a microplate reader. Experiments were performed at 37ºC. CRE (Carbapenem Resistant Enterobacteriaceae) isolates are indicated with a star. Growth curves are shown as the average of 3 separate biological replicates for each microbe and/or microbe/phage pairing with standard deviation bars in each panel.

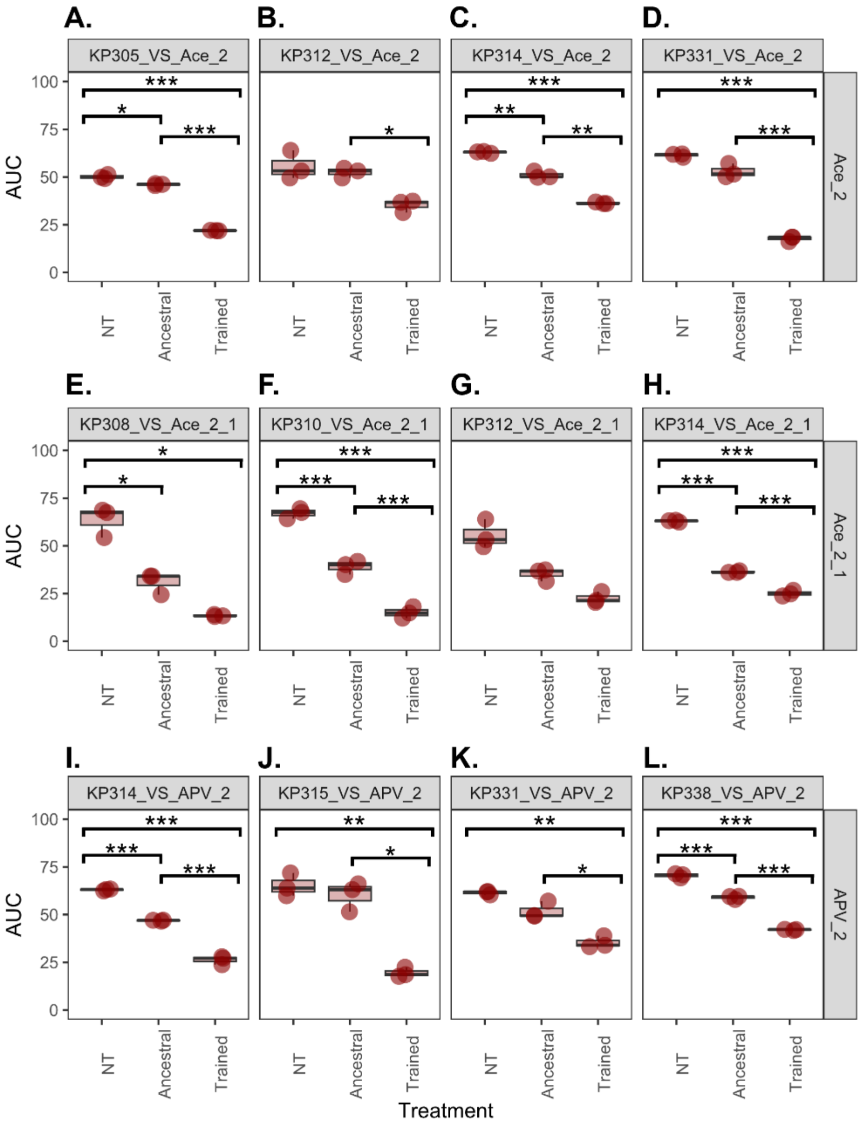

Figure S7. Area Under the Curve (AUC) of growth dynamics of coevolved ESBL isolates grown in presence of trained vs untrained phages at MOI of 1 (Panels A-L). NT represents no treatment, Ancestral represents isolates grown in the presence of the ancestral versions of the phages and Trained represents isolates grown in the presence of the trained phages. Three biological replicates were performed for each experiment. Statistical significance based on AUC analysis is represented by the '*' above in each panel, where the brackets indicate which biological replicates from which experiments are being compared. A single * represents p<0.05, ** represents p<0.01, and *** represents p<0.001.

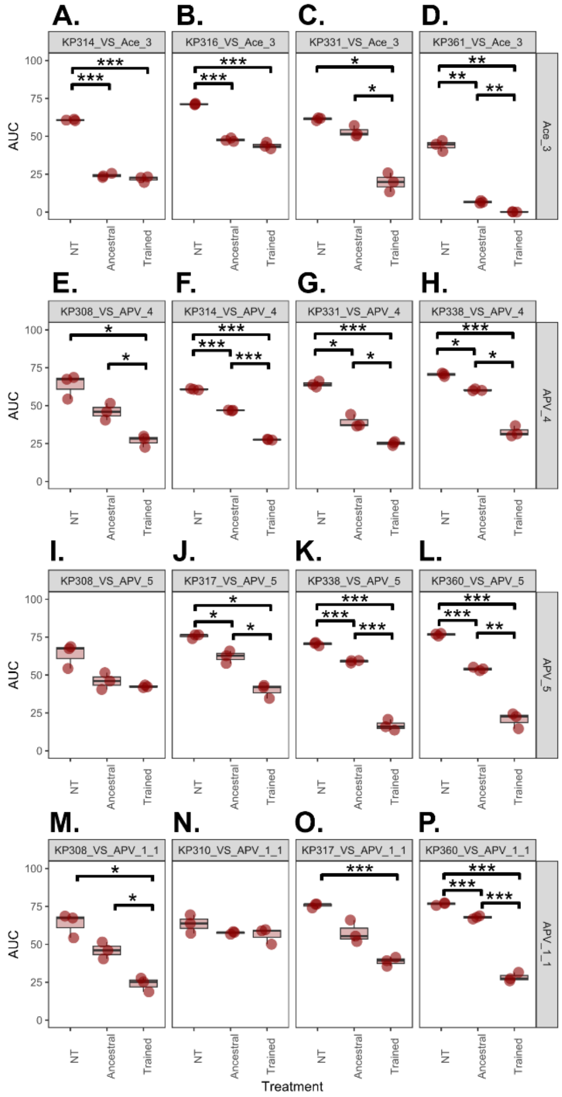

Figure S8. Area Under the Curve (AUC) for growth dynamics of coevolved hosts in presence of trained vs untrained phages at MOI of 1 (Panels A-P). The phage and bacteria were coevolved together for 30 consecutive days, the trained phages after 30 days were isolated and determined their kinetic efficiency on their corresponding coevolved hosts by monitoring bacterial growth for 72 hours at 37℃. The isolates were grouped based on Extended spectrum beta-lactamases (ESBLs). NT represents no treatment, Ancestral represents isolates grown in the presence of the ancestral versions of the phages and Trained represents isolates grown in the presence of the trained phages. Three biological replicates were performed for each experiment. Statistical significance based on AUC analysis is represented by the '*' above in each panel, where the brackets indicate which biological replicates from which experiments are being compared. A single * represents p<0.05, ** represents p<0.01, and *** represents p<0.001.

A)

	ACE	Training strain	Days
ACE.1	99.99	KP96	30
ACE.2	99.99	KP117	30
ACE.2.1	99.99	KP117, KP319	30,30
ACE.3	99.99	KP112	30
ACE.4	99.99	KP122	24
ACE.5	99.99	KP122	30

B)

	APV	Training strain	Days
APV.1	99.99	KP96	30
APV.1.1	99.98	KP96, KP315	30,30
APV.2	99.98	KP117	30
APV.3	99.98	KP112	30
APV.4	99.98	KP112	27
APV.5	99.99	KP315	30

Figure S9. Average Nucleotide Identity between ancestral phage and trained phages for phage Ace A) and phage APV B).

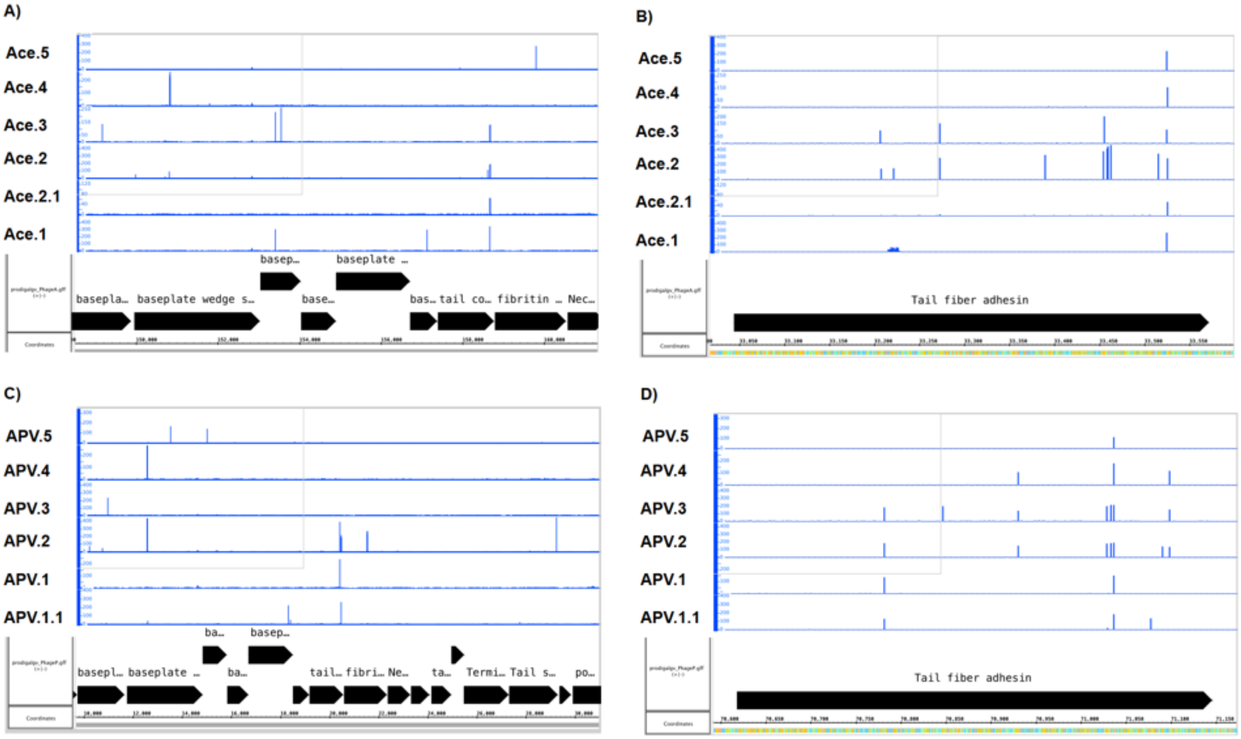

Figure S10. Variants identified in trained phages Ace (A, B) and APV (C, D). Ancestral phages were aligned with trained phage genomes and visualized using the Integrated Genome Browser. The Integrated Genome Browser was then used to generate mismatch graphs from the BAM files showing the number of mismatched nucleotides compared to the reference sequence.

Table S1. List of bacteriophages used in the study and their genome information and isolation source.

Phage	Genome size (bp)	Family	Genus	Isolation source	GenBank ID
LK3	49,048	Drexlerviridae	Webervirus	Excite lab wastewater	PQ621130
LK2	37,934	Autographiviridae	Przondovirus	Excite lab wastewater	PQ621129
Chai	38,369	Autographiviridae	Przondovirus	Excite lab wastewater	PQ621138
APV	176,400	Straboviridae	Slopekvirus	Excite lab wastewater	PQ621122
Ace	175,589	Straboviridae	Slopekvirus	Excite lab wastewater	PQ621123
LK1	177,771	Straboviridae	Slopekvirus	Excite lab wastewater	PQ529758
Beam	170,432	Straboviridae	Jiodavirus	Excite lab wastewater	PQ621133
KL35	165,835	Straboviridae	Jiaodavirus	Excite lab wastewater	PQ621124
QTY	167,428	Straboviridae	Jiaodavirus	Excite lab wastewater	PQ621131
Rec	167,933	Straboviridae	Jiaodavirus	Excite lab wastewater	PQ621132
Turmeric	165,747	Straboviridae	Jiaodavirus	Excite lab wastewater	PQ621121

Table S2. Phages and their top matching closest relatives on NCBI after blastN analysis.

Phages	BLASTn Results	Percent Identity (%)	Query Cover (%)	Genome Size (bp)
LK3	Klebsiella phage vB_Kpn_K52PH129C1 genome assembly, chromosome: K52PH129C1_assembly	95.71	90	49,703
	Klebsiella phage KPN N141, complete genome	97.07	88	49,090
	Klebsiella phage vB_KpnS-VAC70, complete genome	95.79	90	49,631
LK2	Klebsiella phage vB_KpnP_ZX10, complete genome	96.48	94	39,301
	Klebsiella phage vB_KpI_K8PH128 genome assembly, chromosome: K8PH128_assembly	95.88	98	40,663
	Klebsiella phage K5-4, complete genome	93.42	91	40,163
Chai	Klebsiella phage vB_KpI_K8PH128 genome assembly, chromosome: K8PH128_assembly	97.50	99	40,663
	Klebsiella phage 2044-307w, complete genome	94.29	95	40,048
	Klebsiella phage KP32_isolate 196, complete genome	94.27	85	40,037
APV	Klebsiella phage PSKm2DI, complete genome	98.69	97	177,720
	Klebsiella phage mtp7 genome assembly, chromosome: 1	98.55	95	177,726
	Klebsiella phage vB_KoM-Liquor genome assembly, chromosome: 1	98.67	96	176,734
Ace	Klebsiella phage Matisse, complete genome	99.03	98	176,081
	Klebsiella phage AYL, complete genome	99.15	98	175,000
	Klebsiella phage Miro, complete genome	99.00	97	176,005
LK1	Klebsiella phage phiKp_19 DNA, complete genome	98.96	95	176,015
	Klebsiella phage phiKp_12 DNA, complete genome	98.95	95	176,058
	Klebsiella phage phiKp_11 DNA, complete genome	98.96	95	176,058
Beam	Klebsiella phage R3_1, complete genome	98.92	97	169,297
	Klebsiella phage vB_Kpn_P545, complete genome	99.10	96	169,275
	Klebsiella phage PhiKpNIH-6, complete genome	98.03	96	171,860
KL35	Klebsiella phage pzk-kv19, complete genome	96.14	97	167,127
	Klebsiella phage vB_KpnM_FRZ284, complete genome	97.14	98	166,376
	Klebsiella phage vB_KpnM_311F genome assembly, chromosome: 1	97.18	97	166,710
QTY	Klebsiella pneumoniae phage strain 5899STDY8049226 genome assembly, chromosome: 1	97.00	96	165,752
	Klebsiella phage JD18, complete genome	96.99	95	166,313
	Klebsiella phage pKp20, complete genome	96.70	95	165,762
Rec	Klebsiella phage ValerieMcCarty03, complete genome	97.00	94	168,196
	Klebsiella phage phiKp_22 DNA, complete genome	96.76	94	167,801
	Klebsiella pneumoniae phage JIPh_Kp122, complete genome	96.26	96	166,475
Turmeric	Klebsiella phage UTI-K4, complete genome	95.71	98	165,805
	Klebsiella phage vB_KpnM_FRZ284, complete genome	98.56	96	166,376
	Salmonella phage PSE-D1, complete genome	97.47	97	166,604

Table S3. List of bacterial strains, their antibiotic resistance profile and isolation source. The antibiotics were named using the EUCAST system.

Klebsiella strains	Species	ESBL/ Non ESBL	Resistant Antibiotics	Isolation source
KP96	*K. pneumoniae*	Non ESBL	AMP, CXI, AMS	body site kidney
KP98	*K. pneumoniae*	Non ESBL	AMP, NIT	urine
KP99	*K. pneumoniae*	Carbapenem	CTZ, CIP, CTR, CEP, GEN, TOB, AMS, TRS, AMP, CZO, CXI, PIT, ERT	sputum
KP101	*K. pneumoniae*	Non ESBL	AMP	sputum
KP103	*K. pneumoniae*	Non ESBL	AMP, NIT	urine
KP104	*K. pneumoniae*	Non ESBL	CTZ, CIP, CTR,CZO, CEP, NIT, CXI, GEN, TOB, AMS, TRS, PIT, AMP	urine
KP106	*K. pneumoniae*	Non ESBL	AMP	drainage hip
KP111	*K. oxytoca*	Non ESBL	AMP	abscess drain
KP112	*K. pneumoniae*	Non ESBL	AMP, CZO, CXI, AMS	bronch wash
KP114	*K. pneumoniae*	Non ESBL	AMP	bronch wash
KP117	*K. pneumoniae*	Non ESBL	-	urine
KP118	*K. pneumoniae*	Non ESBL	AMP, CXI	urine
KP122	*K. pneumoniae*	Non ESBL	-	urine
KP124	*K. aerogenes*	Non ESBL	AMP	body site (aka E.
KP127	*K. pneumoniae*	Non ESBL	AMP, CTZ, CIP, CTR, CZO, CEP, NIT, GEN, TOB, AMS, TMS, PIT	urine
KP129	*K. pneumoniae*	Non ESBL	AMP, CZO	blood
KP130	*K. pneumoniae*	Non ESBL	AMP	urine
KP134	*K. aerogenes*	Non ESBL	AMP, CZO, NIT, AMS	urine
KP135	*K. pneumoniae*	Non ESBL	AMP	Urine
KP137	*K. pneumoniae*	Non ESBL	AMP, CIP, TMS	urine
KP301	*K. pneumoniae*	ESBL	AMP, AZT, CTZ, CIP, CTR, CZO, CEP, AMS, TMS, PIT	Unknown
KP302	*K. pneumoniae*	ESBL	AMP, CTZ, CIP, CTR, CZO, CEP, AMS, AMS, PIT	Urine
KP303	*K. pneumoniae*	ESBL	AMP, CTZ, CIP, CTR, CZO, CEP, GEN, AMS, TMS, PIT	Bronchial Washings
KP304	*K. pneumoniae*	ESBL	AMP, CTZ, CIP, CTZ, CZO, CEP, AMS, PIT	Abscess
KP305	*K. pneumoniae*	ESBL	AMP, CTZ, CIP, CTR, CZO, CEP, GEN, TOB, AMS, TMS, PIT	Sputum
KP306	*K. pneumoniae*	ESBL	AMP, AZT, CTZ, CIP, CTR, CZO, CEP, GEN, AMS, TMS, PIT	Unknown
KP307	*K. pneumoniae*	Carbapenem	AMP, CTZ, CTR, CZO, CEP, AMS, PIT, CTT, MIN, ERT, MER	Abscess
KP308	*K. pneumoniae*	ESBL	AMP, CTZ, CIP, CTR, CZO, CEP, AMS, TMS, PIT	Urine
KP309	*K. pneumoniae*	ESBL	AMP, CTZ, CIP, CTR, CZO, CEP, AMS, TMS, PIT	Urine
KP310	*K. pneumoniae*	Carbapenem	AMP, CTZ, CIP, CTR, CZO, CEP, GEN, TOB, AMS, TMS, PIT, AZT, LEV, TET, CTT,	Blood
KP311	*K. pneumoniae*	ESBL	AMP, AMI, CTZ, CIP, CTR, CZO, CEP, NIT, GEN, TOB, AMS, TMS, PIT, CTT, CTV,	Urine
KP312	*K. pneumoniae*	Carbapenem	AMP, AMI, CTZ, CIP, CTR, CZO, CEP, GEN, TOB, AMS, TMS, PIT, CTT, CTV, MER,	Respiratory
KP313	*K. pneumoniae*	ESBL	AMP, AMI, CTZ, CIP, CTR, CZO, CEP, GEN, TOB, AMS, TMS, PIT, CTT, CTV	Sputum
KP314	*K. pneumoniae*	ESBL	AMP, CTZ, CIP, CTR, CZO, CEP, GEN, TOB, AMS, TMS, PIT, CTT, MIN	Tracheal Aspirate
KP315	*K. pneumoniae*	ESBL	AMP, CTZ, CIP, CTR, CZO, CEP, AMS, TMS, PIT	Urine
KP316	*K. pneumoniae*	ESBL	AMP, CTZ, CIP, CTR, CZO, CEP, AMS, TMS, PIT, FOS	Urine
KP317	*K. pneumoniae*	ESBL	AMP, CTZ, CIP, CTR, CZO, CEP, NIT, AMS, TMS, PIT, FOS	Urine
KP318	*K. pneumoniae*	ESBL	AMP, AZT, CTZ, CIP, CTR, CZO, CEP, GEN, LEV, TOB, AMS, TMS	Unknown
KP319	*K. pneumoniae*	ESBL	AMP, CTZ, CTR, CZO, CEP, GEN, TOB, AMS, PIT	Tracheal Aspirate
KP320	*K. pneumoniae*	ESBL	AMP, CTZ, CIP, CTR, CZO, CEP, GEN, AMS, TMS, PIT	Urine
KP321	*K. pneumoniae*	ESBL	AMP, CTZ, CIP, CTR, CZO, CEP, NIT, GEN, TOB, AMS,TMS, PIT	Urine
KP328	*K. pneumoniae*	ESBL	AMP, CTZ, CIP, CTR, CZO, CEP, GEN, AMS, TMS, PIT, FOS	Urine
KP329	*K. pneumoniae*	ESBL	AMP, CTZ, CIP, CTR, CZO, CEP, AMS, TMS, PIT	Urine
KP331	*K. pneumoniae*	ESBL	AMP, CTZ, CIP, CTR, CZO, CEP, AMS, TMS, PIT	Urine
KP332	*K. pneumoniae*	ESBL	AMP, CTZ, CIP, CTR, CZO, NIT, AMS, TMS, PIT	Urine
KP333	*K. pneumoniae*	ESBL	AMP, CTZ, CTR, CZO, CEP, NIT, AMS, PIT	Urine
KP334	*K. pneumoniae*	Non ESBL	AMP	Urine
KP337	*K. pneumoniae*	ESBL	AMP, CTZ, CIP, CTR, CZO, CEP, AMS, TMS, PIT	Respiratory - Cystic
KP338	*K. pneumoniae*	ESBL	AMP, CTZ, CIP, CTR, CZO, CEP, GEN, TOB, AMS, TMS, PIT	Swab for Culture
KP339	*K. pneumoniae*	ESBL	AMP, CTZ, CIP, CTR, CZO, CEP, GEN, TOB, AMS, TMS, PIT	Urine
KP340	*K. pneumoniae*	ESBL	AMP, CTZ, CIP, CTR, CZO, CEP, GEN, TOB, AMS, TMS, PIT	Abscess
KP341	*K. pneumoniae*	ESBL	AMP, AMI, CTZ, CIP, CTR, CZO, CEP, GEN, TOB, AMS, TMS, PIT	Respiratory - Cystic
KP342	*K. pneumoniae*	ESBL	AMP, CTZ, CIP, CTR, CZO, CEP, GEN, AMS, TMS, PIT	Lesion
KP343	*K. pneumoniae*	ESBL	AMP, CTZ, CIP, CTR, CZO, CEP, AMS, PIT	Bronchial Washings
KP344	*K. pneumoniae*	ESBL	AMP, CTZ, CIP, CTR, CZO, CEP, GEN, TOB, AMS,TMS, PIT	Sputum
KP345	*K. pneumoniae*	ESBL	AMP, CTZ, CIP, CTR, CZO, CEP, AMS, TMS, PIT	Urine
KP347	*K. pneumoniae*	ESBL	AMP, CTZ, CIP, CTR, CZO, CEP, AMS, PIT, FOS	Urine
KP346	*K. pneumoniae*	ESBL	AMP, CTZ, CIP, CTR, CZO, CEP, NIT, AMS, TMS, PIT	Urine
KP360	*K. pneumoniae*	ESBL	AMP, AZT, CTZ, CIP, CTR, CZO, CEP, GEN, TET, TOB, AMS, TMS, PIT	Unknown
KP361	*K. pneumoniae*	Carbapenem	AMI, AMP, AZT, CTZ, CIP, CTR, CZO, CEP, ERT, LEV, TOB, AMS, TMS, PIT	Unknown
KP358	*K. pneumoniae*	ESBL	AMP, CTZ, CIP, CTR, CZO, CEP, NIT, AMS, PIT, FOS	Urine

Table S4: CheckV and assembly results.

Contig_id	Contig_length	Provirus	Gene_count	Viral_genes	Host_genes	Checkv_quality	Miuvig_quality	Completeness	Completeness_method	Contamination	kmer_freq
LK3	49048	No	72	66	0	High-quality	High-quality	99	AAI-based (high-confidence)	0	1
LK2	37934	No	43	41	0	High-quality	High-quality	94.64	AAI-based (high-confidence)	0	1
Chai	38369	No	43	42	0	High-quality	High-quality	95.72	AAI-based (high-confidence)	0	1
APV	176400	No	277	194	0	High-quality	High-quality	99.01	AAI-based (high-confidence)	0	1
Ace	175589	No	273	193	0	High-quality	High-quality	98.56	AAI-based (high-confidence)	0	1
LK1	177771	No	274	192	0	High-quality	High-quality	99.78	AAI-based (high-confidence)	0	1
Beam	170432	No	283	230	0	High-quality	High-quality	99.05	AAI-based (high-confidence)	0	1
KL35	165835	No	262	236	0	High-quality	High-quality	98.23	AAI-based (high-confidence)	0	1
QTY	123255	No	180	165	0	Medium-quality	Genome-fragment	73.02	AAI-based (high-confidence)	0	1
Rec	167933	No	265	238	0	High-quality	High-quality	99.34	AAI-based (high-confidence)	0	1
Turmeric	165747	No	262	236	0	High-quality	High-quality	98.17	AAI-based (high-confidence)	0	1
