# Supplementary material for: Experimental phage evolution results in expanded host ranges against antibiotic resistant Klebsiella pneumoniae isolates
Source: Nat Commun. 2025 Nov 19;16:9903. doi: 10.1038/s41467-025-66062-7 (PMC12630596; doi:10.1038/s41467-025-66062-7)
Supplement: Supplementary file 2 — Reporting Summary [file 41467_2025_66062_MOESM2_ESM.pdf]

nature portfolio

Corresponding author(s): David T. Pride

Last updated by author(s): 2025/10/17

Reporting Summary

Nature Portfolio wishes to improve the reproducibility of the work that we publish. This form provides structure for consistency and transparency in reporting. For further information on Nature Portfolio policies, see our Editorial Policies and the Editorial Policy Checklist.

Statistics

For all statistical analyses, confirm that the following items are present in the figure legend, table legend, main text, or Methods section.

n/a	Confirmed	
☐	☒	The exact sample size (*n*) for each experimental group/condition, given as a discrete number and unit of measurement
☐	☒	A statement on whether measurements were taken from distinct samples or whether the same sample was measured repeatedly
☐	☒	The statistical test(s) used AND whether they are one- or two-sided<br>*Only common tests should be described solely by name; describe more complex techniques in the Methods section.*
☒	☐	A description of all covariates tested
☒	☐	A description of any assumptions or corrections, such as tests of normality and adjustment for multiple comparisons
☒	☐	A full description of the statistical parameters including central tendency (e.g. means) or other basic estimates (e.g. regression coefficient) AND variation (e.g. standard deviation) or associated estimates of uncertainty (e.g. confidence intervals)
☒	☐	For null hypothesis testing, the test statistic (e.g. *F*, *t*, *r*) with confidence intervals, effect sizes, degrees of freedom and *P* value noted<br>*Give P values as exact values whenever suitable.*
☒	☐	For Bayesian analysis, information on the choice of priors and Markov chain Monte Carlo settings
☒	☐	For hierarchical and complex designs, identification of the appropriate level for tests and full reporting of outcomes
☒	☐	Estimates of effect sizes (e.g. Cohen's *d*, Pearson's *r*), indicating how they were calculated

Our web collection on statistics for biologists contains articles on many of the points above.

Software and code

Policy information about availability of computer code

Data collection	N/A.
Data analysis	The code used in this work are publicly available on GitHub (https://github.com/raw-lab/) and at https://doi.org/10.5281/zenodo.17296047.

For manuscripts utilizing custom algorithms or software that are central to the research but not yet described in published literature, software must be made available to editors and reviewers. We strongly encourage code deposition in a community repository (e.g. GitHub). See the Nature Portfolio guidelines for submitting code & software for further information.

Data

Policy information about availability of data

All manuscripts must include a data availability statement. This statement should provide the following information, where applicable:

- Accession codes, unique identifiers, or web links for publicly available datasets
- A description of any restrictions on data availability
- For clinical datasets or third party data, please ensure that the statement adheres to our policy

All phage genomes used in this study are available on NCBI GenBank (PQ621130, PQ621129, PQ621128, PQ621122, PQ621123, PQ529758, PQ621133, PQ621124, PQ621131, PQ621132, PQ621121).

Research involving human participants, their data, or biological material

Policy information about studies with human participants or human data. See also policy information about sex, gender (identity/presentation), and sexual orientation and race, ethnicity and racism.

Reporting on sex and gender	Research did not directly involve human subject participation. It involved bacteria derived from human subjects. Sex and gender were not reported.
Reporting on race, ethnicity, or other socially relevant groupings	Research did not directly involve human subject participation. It involved bacteria derived from human subjects. Sex and gender were not reported.
Population characteristics	Because the research did not directly involve human subjects, there was no reporting on population characteristics.
Recruitment	There was no human subject recruitment.
Ethics oversight	All bacterial isolates collected in this study were approved by the University of California San Diego Administrative Panel on Human Subjects in Medical Research. This Study was categorized as exempt, which does not require informed consent on behalf of the study subjects. It was categorized as exempt because it involved the use of existing records and specimens that were recorded in such a manner that the subjects could not be identified directly or through identifiers linked to the subjects.

Note that full information on the approval of the study protocol must also be provided in the manuscript.

Field-specific reporting

Please select the one below that is the best fit for your research. If you are not sure, read the appropriate sections before making your selection.

[x] Life sciences [ ] Behavioural & social sciences [ ] Ecological, evolutionary & environmental sciences

For a reference copy of the document with all sections, see nature.com/documents/nr-reporting-summary-flat.pdf

Life sciences study design

All studies must disclose on these points even when the disclosure is negative.

Sample size	We tested a collection of 11 different Klebsiella bacteriophages against 59 different Klebsiella clinical isolates. We then evolved 2 of those phages with moderate activity against the clinical isolates over 30 day time periods to improve their host ranges. We tested different recovered phages from these experiments.
Data exclusions	We excluded Klebsiella isolates that had inducible prophages because those prophages likely would have intefered with the results of the experiments.
Replication	Each of the experiments in the study were performed in triplicate and results shown with means and standard deviations, and areas under the curves to determine whether there is statistical significance to the inhibition observed with trained phages compared to ancestral phages.
Randomization	There was no randomization scheme developed.
Blinding	N/A.

Reporting for specific materials, systems and methods

We require information from authors about some types of materials, experimental systems and methods used in many studies. Here, indicate whether each material, system or method listed is relevant to your study. If you are not sure if a list item applies to your research, read the appropriate section before selecting a response.

Materials & experimental systems

n/a	Involved in the study	
[x]	[ ]	Antibodies
[x]	[ ]	Eukaryotic cell lines
[x]	[ ]	Palaeontology and archaeology
[x]	[ ]	Animals and other organisms
[ ]	[x]	Clinical data
[ ]	[x]	Dual use research of concern
[x]	[ ]	Plants

Methods

n/a	Involved in the study	
[x]	[ ]	ChIP-seq
[x]	[ ]	Flow cytometry
[x]	[ ]	MRI-based neuroimaging

Clinical data

Policy information about clinical studies

All manuscripts should comply with the ICMJE guidelines for publication of clinical research and a completed CONSORT checklist must be included with all submissions.

Clinical trial registration	N/A
Study protocol	N/A
Data collection	N/A
Outcomes	N/A

Dual use research of concern

Policy information about dual use research of concern

Hazards

Could the accidental, deliberate or reckless misuse of agents or technologies generated in the work, or the application of information presented in the manuscript, pose a threat to:

No	Yes	
☒	☐	Public health
☒	☐	National security
☒	☐	Crops and/or livestock
☒	☐	Ecosystems
☒	☐	Any other significant area

Experiments of concern

Does the work involve any of these experiments of concern:

No	Yes	
☒	☐	Demonstrate how to render a vaccine ineffective
☒	☐	Confer resistance to therapeutically useful antibiotics or antiviral agents
☒	☐	Enhance the virulence of a pathogen or render a nonpathogen virulent
☒	☐	Increase transmissibility of a pathogen
☒	☐	Alter the host range of a pathogen
☒	☐	Enable evasion of diagnostic/detection modalities
☒	☐	Enable the weaponization of a biological agent or toxin
☒	☐	Any other potentially harmful combination of experiments and agents

Plants

Seed stocks	N/A
Novel plant genotypes	N/A
Authentication	N/A
